# Using Primary Care Parenting Interventions to Improve Outcomes in Children with Developmental Disabilities: A Case Report

**DOI:** 10.1155/2012/150261

**Published:** 2012-08-13

**Authors:** Cassandra L. Tellegen, Matthew R. Sanders

**Affiliations:** Parenting and Family Support Centre, Social Sciences Building, University of Queensland, St Lucia, QLD 4072, Australia

## Abstract

Parenting is central to the health and well-being of children. Children with developmental disabilities have been shown to be at increased risk of developing emotional and behavioral problems. Parent training programs are effective interventions for improving child behavior and family functioning. This paper describes the outcomes of a brief 4-session parenting intervention (Primary Care Stepping Stones Triple P) targeting compliance and cooperative play skills in an 8-year-old girl with Asperger's disorder and ADHD combined type. The intervention was associated with decreases in child behavior problems, increases in parenting confidence, and decreases in dysfunctional parenting styles. This paper demonstrates that low-intensity parenting interventions can lead to significant improvements in child behavior and family functioning. Such brief interventions are cost effective, can be widely disseminated, and have been designed to be delivered within primary health care settings. Pediatricians can play a key role in identifying parents in need of assistance and in helping them access evidence-based parenting interventions.

## 1. Introduction

Children with developmental disabilities are at increased risk of developing behavioral and emotional problems [1]. Parenting practices (e.g., quality of parenting and harsh disciplinary practices) and family factors (e.g., family dysfunction and parental distress) are important risk factors in the development of behavioral and emotional problems in children with disabilities [2]. Furthermore, parenting programs have been shown to improve parent and child outcomes in families with a child with disability [3]. Hence the provision of parenting programs for parents of children with disabilities is warranted. Due to their ongoing contact with families, pediatricians have an important role to play in promoting and disseminating parenting services [4].

Pediatricians are well placed to identify families in need of parenting support and to either provide services within their practice or recommend outside services. An Australian study profiling the content of pediatric consultations reported that almost half of all patients were presenting with ongoing child behavioral difficulties [5]. A recent policy statement from the American Academy of Pediatrics [6] emphasized the need for pediatricians to adopt a more proactive leadership role in promoting the implementation of interventions which support healthy child development, such as positive parenting programs.

One system of evidence-based parenting interventions for children with disabilities is Stepping Stones Triple P. Stepping Stones Triple P is a parallel version of the Triple P-Positive Parenting Program which aims to prevent and treat childhood behavioral and emotional problems by enhancing the skills, confidence, and knowledge of parents [7]. A large body of research supports the efficacy and effectiveness of Triple P, including four independent meta-analyses which have demonstrated improvements in child behavior problems and parenting practices [8–11].

Stepping Stones Triple P takes a public health perspective to promote widespread dissemination of parenting programs and provide the minimally sufficient level of intervention required to meet the needs of each individual family [12]. The Stepping Stones Triple P system consists of a range of parenting programs which vary according to the amount of contact with practitioners and delivery format. Randomized controlled trials have provided evidence for the efficacy of Stepping Stones Triple P when presented in a seminar format [13], and when delivered as 8–10 group or individual sessions [14–16]. A trial is currently evaluating the efficacy of a brief, individually-delivered version of Stepping Stones Triple P: Primary Care Stepping Stones Triple P (PCSSTP) [17].

PCSSTP consists of four brief sessions targeting one or two specific child problems. This program is designed to be delivered by primary health care providers and allied staff, including pediatricians and child health nurses. PCSSTP was developed for parents of children with a disability who are presenting with relatively discrete concerns about their child's behavior or development. This paper describes the treatment and outcomes for the family of an 8-year-old girl with Asperger's disorder and attention deficit hyperactivity disorder (ADHD) combined type whose parents took part in a brief parenting intervention.

## 2. Case Presentation

Jan (aged 38) and Frank (aged 40), a married couple, attended parenting sessions to receive help with managing difficult behaviors in their 8-year-old daughter, Zoe. Zoe was diagnosed by her pediatrician with Asperger's disorder and ADHD combined type. Zoe had one younger brother, and attended a mainstream primary school. Zoe's parents did not access any other services while participating in the program. Jan and Frank described Zoe as high functioning, with good communication, and self-help skills. Zoe's parents reported that she had difficulties interacting in social situations, and difficulties with fine motor skills and hand-eye coordination. Jan and Frank were seeking assistance for two main concerns with Zoe's behavior: her noncompliance and her cooperative play skills.

Zoe's parents reported that she was noncompliant with instructions during 2–5 incidents per day. For example, she would not comply with instructions to get out of bed, get dressed, or to help with chores, unless instructions were repeated a number of times. Jan and Frank were also concerned about Zoe's cooperative play skills. They reported that when playing games with others such as soccer or board games, Zoe would not follow the rules of the game or would change the rules without telling others. Her parents reported that they found playing games with her frustrating and as a result were spending less time playing with her. Jan and Frank were concerned that if Zoe's cooperative play skills did not improve it would prevent her developing relationships with her peers.

Initial assessment involved an intake interview during the first session of the program and the completion of standardized questionnaires by Jan. A number of important maintenance factors contributing to Zoe's behavior problems were identified by Jan and Frank: accidental positive reinforcement for misbehavior; poorly timed instructions; repetition of instructions; parents raising their voice and escalating to achieve compliance; inconsistently applied consequences or lack of consequences for misbehavior; parents' avoiding play activities with Zoe.

Jan completed a series of standardized, well-validated questionnaires before starting the program, following program completion, and 6 months later. Jan's scores on the Eyberg Child Behaviour Inventory (ECBI) [18], indicated that Zoe's behavior was in the clinical range in terms of the both the frequency of behavior problems and the number of problems experienced. Jan reported 26 out of 36 child behaviors as being consistent difficulties. On the Parenting Scale [19], Jan scored in the clinical range for the overreactivity and verbosity subscales indicating clinical levels of these dysfunctional parenting styles. Jan's scores on the Parent Problem Checklist (PPC) [20] and the Relationship Quality Inventory (RQI) [21] were in the nonclinical range, indicating very high levels of relationship satisfaction and low levels of marital disagreement. Jan's scores on the Depression Anxiety Stress Scales (DASS-21) [22] indicated minimal symptoms of depression, anxiety, and stress. Her scores on the Parental Stress Scale [23] indicated elevated levels of parenting-related stress. Jan's responses on the Parenting Tasks Checklist [24] indicated high levels of confidence in dealing with difficult child behaviors in a range of different settings. However, her confidence in dealing with a range of difficult child behaviors fell below the clinical cut-off for this subscale.

Jan and Frank participated in Primary Care Stepping Stones Triple P, which involved four individual sessions spanning a 7-week period. The practitioner was a provisional psychologist who followed a manualized protocol. The first session was primarily an intake interview to collect family background information and discuss presenting problems. Jan and Frank selected two child behaviors to target during the program, and monitored these behaviors the following week. Parents were given a booklet outlining the Stepping Stones Triple P principles and strategies. The second session involved a review of the assessment information; discussion of the monitoring and parents' perceptions of the problem; sharing conclusions about the nature and etiology of the problem; setting goals for change; and developing a parenting plan. Two additional Stepping Stones Triple P booklets providing information on strategies for addressing the two target behavior problems (disruptive behavior and social skills) were given to the parents. Session 3 included a review of progress and making adjustments to the parenting plan. Session 4 involved a review of progress in terms of set goals; discussion of any obstacles encountered; termination of contact. The couple was motivated to participate in the treatment, attended sessions regularly, and completed assigned homework tasks.

**Figure 1:**
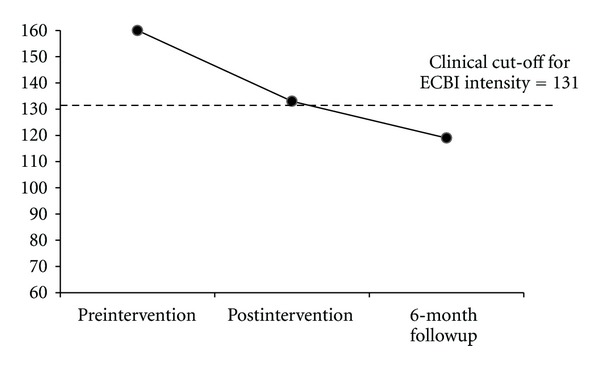
Change on ECBI intensity scale at preintervention, postintervention, and 6-month followup.

The success of the program was evaluated in terms of changes on child and parent outcomes across the intervention and at 6-month followup. Child behavior problems decreased significantly over time and Zoe was moved out of the clinical range on both scales of the ECBI.  Figure 1 displays the improvement in the frequency of child behavior problems over the three measurement occasions.

Jan's use of dysfunctional parenting strategies decreased reliably from preintervention to postintervention. In particular, her scores on the over-reactivity and verbosity subscales of the Parenting Scale moved from the clinical to normal range. While Jan's confidence in dealing with child behaviors across a range of settings remained high across all time points, her confidence in dealing with difficult child behaviors was low at preintervention and improved considerably. Jan remained in the normal range for reported levels of parental conflict (PPC), relationship satisfaction (RQI), and depression and stress (DASS-21). However, Jan's anxiety symptoms deteriorated from postintervention to followup; Jan moved from the normal to the mild range for anxiety symptoms on the DASS-21.  Table 1 displays the scores on all measures at three time points indicating whether each score improved by moving out of the clinical range (clinical ranges were based on published clinical cut-offs or were calculated using the formula recommended by Jacobson and Truax [25]), remained in the normal range, or deteriorated.

Jan and Frank also completed the Goal Achievement Scales [26]. These scales allow for an estimate of the percentage of success attained in reaching specific goals. During the second session, goals for change over the intervention period were set for the two target child behaviors: compliance and cooperative play skills. In reference to baseline monitoring data collected during the first week of intervention, Jan and Frank indicated the rate of each target behavior that would be ideal to reach by the end of the four sessions. At the fourth session, monitoring data indicated that both goals had been met with 75% success. At program completion, both parents reported high levels of satisfaction with the program.

## 3. Discussion

This case highlights the benefits that primary care parenting interventions can have on family functioning. The parents of an 8-year-old girl with Asperger's disorder and ADHD combined type participated in a four-session parenting program which led to significant reductions in child behavior problems, improvements in parenting confidence, and decreases in the use of dysfunctional parenting styles. These improvements continued or were maintained over a 6-month period following completion of the brief program. Moreover, the parents reported high rates of success in achieving their specific goals for change in child behavior. No significant improvements in parental distress, parental conflict, or relationship satisfaction were achieved due to this family functioning in the healthy range on these measures before beginning the program.

Parenting and family functioning is central to the well-being of children. It is evident that low-intensity parenting interventions can lead to significant and meaningful changes in families with complex presentations, challenging the notion that complex problems require complex solutions. Further research evaluating this intervention in controlled studies with a broad range of families is needed to support widespread dissemination of this parenting program. Additionally, while follow-up results suggested that outcomes were sustainable, evaluation of maintenance effects over longer time periods (e.g., 12 months) is recommended.

Brief interventions are cost-effective, time-efficient, and have the capacity to be widely disseminated through primary health care services. Low-intensity parenting interventions are best suited to parents who require assistance for discrete child problems, are motivated to participate in sessions, and are not currently impaired by parental psychopathology. Interventions such as Stepping Stones Triple P promote self-sufficiency and focus on training parents to generalize parenting skills so that they are better equipped to successfully deal with difficult child problems which may arise in the future. As a main point of contact for parents of children with disabilities, it is important that pediatricians recognize the value of parenting interventions. Pediatricians have a key role to play in promoting child health by identifying families in need of assistance and by helping them to access effective parenting services.

## Figures and Tables

**Table 1:** Scores on each of the measures at three time points and indication of whether scores showed improvement by moving out of the clinical range.

Measure	Time 1: Preintervention	Time 2: Postintervention	Time 3: 6-month followup	Showed improvement by moving out of the clinical range?
ECBI intensity	160^∗^	133^∗^	119	Improved
ECBI problem	26^∗^	15^∗^	13	Improved
PS laxness	2.45	2.09	2.09	Remained in normal range
PS overreactivity	4.2^∗^	2.00	1.7	Improved
PS verbosity	4.57^∗^	3.43	1.57	Improved
PPC problem	2	2	3	Remained in normal range
PPC extent	24	20	25	Remained in normal range
RQI	45	45	44	Remained in normal range
PSS total	40^∗^	35	39	Improved
Depression	2	6	2	Remained in normal range
Anxiety	0	0	8^∗^	Deteriorated
Stress	10	4	6	Remained in normal range
PTC setting	93	95	94	Remained in normal range
PTC behavior	62^∗^	93	93	Improved

^∗^Score falls in clinical range. ECBI: Eyberg Child Behaviour Inventory, PS: Parenting Scale, PPC: Parent Problem Checklist; RQI: Relationship Quality Inventory; PSS: Parental Stress Scale; PTC: Parenting Tasks Checklist.
